# Tumor-Promoting Gut Microbes in Colorectal Cancer: Mechanisms and Translational Perspectives

**DOI:** 10.7150/ijms.123494

**Published:** 2026-01-01

**Authors:** Yulong Yu, Weiheng Zhao, Mu Yang, Bili Wu, Xianglin Yuan

**Affiliations:** Department of Oncology, Tongji Hospital, Tongji Medical College, Huazhong University of Science and Technology, Wuhan, Hubei, China.

**Keywords:** colorectal cancer, gut microbes, therapeutic strategies, tumor immunology

## Abstract

Colorectal cancer (CRC) represents a predominant global malignancy, characterized by increasing incidence and mortality rates. Recent investigations have underscored the gut microbiota as a pivotal element in the pathogenesis and progression of CRC. This review synthesizes current evidence regarding the association between gut microbial dysbiosis and CRC, with a particular emphasis on pathogenic bacteria such as *Fusobacterium nucleatum, enterotoxigenic Bacteroides fragilis, pks⁺ Escherichia coli,* and *Enterococcus faecalis*, among others. The mechanisms through which these microbes contribute to tumorigenesis include the induction of DNA damage, the promotion of chronic inflammation, and the induction of immunosuppression, and the production of oncogenic metabolites. Additionally, the review examines the clinical implications of gut microbiota, highlighting their potential as non-invasive biomarkers for early CRC detection and their impact on the efficacy and toxicity of chemotherapy, radiotherapy, and immunotherapy. Furthermore, emerging microbiota-targeted interventions, such as fecal microbiota transplantation, dietary modification, and probiotics, are evaluated for their therapeutic potential. Despite substantial progress, challenges remain in standardizing microbial markers and optimizing individualized microbiota modulation strategies. Future studies integrating multi-omics and machine learning approaches may pave the way for microbiome-based precision medicine in CRC.

## 1. Background

Cancer is one of the major global public health challenges. The incidence of cancer has been rising due to environmental changes, dietary habits, lifestyle factors, and population aging. This increase poses a significant threat to human health and results in substantial economic losses^1^. Colorectal cancer is a common and highly malignant tumor, ranking third globally in incidence and second in mortality among all cancer types. The latest statistics estimate that, in 2022, there were approximately 1.9 million new cases of colorectal cancer and 0.9 million related deaths worldwide^2^. The development of colorectal cancer is influenced by multiple factors, including age, gender, lifestyle, obesity, diet, and environmental conditions^3^-^7^. Recent research has increasingly highlighted the role of gut microbiota in the onset and progression of colorectal cancer^8^.

Gut microbes are microbial communities that reside in the human gut, along with the gut environment, forming the gut microbiome. Literature reports that the human gut hosts trillions of microbial cells from over a thousand species, including bacteria, fungi, archaea, protists, and viruses, with bacteria being the most abundant^9^. The vast and complex gut microbiota contains a collective microbial genome far larger than the human genome, encoding over 3 million genes, often referred to as the “second genome” of the human body^10^, ^11^. Certain strains of gut microbiota play key roles in digestion, the production of beneficial metabolites, immunity regulation, and defense against pathogenic microorganisms. An imbalance in the gut microbiome can lead to digestive disorders, including ulcerative colitis, Crohn's disease, and irritable bowel syndrome^12^-^14^. The majority of human gut microbiota resides in the colon, the most common site for digestive tract tumors. Studies have shown that colorectal cancer patients exhibit significant alterations in their gut microbiota^15^. Compared to healthy individuals, these patients have marked differences in species composition and microbial abundance, including an increased abundance of cancer-associated microbes and a decrease in the abundance of protective microbes^16^. These suggest that imbalances in gut microbiota composition may be closely associated with colorectal cancer development. However, it remains unclear whether the alterations in gut microbiota are a cause or a consequence of colorectal carcinogenesis. The interaction between gut microbiota and colorectal cancer has become a prominent research topic in recent years.

Advances in genome sequencing and bioinformatics, particularly the development of 16S rRNA gene sequencing and metagenomic sequencing technologies, have revolutionized scientific research. These innovations have significantly enhanced our ability to study complex gut microbiota, improving the identification of intestinal microorganisms and enabling deeper exploration of the relationship between gut microbiota and tumors. This paper reviews recent research on gut microbiota and colorectal cancer, analyzing future research directions to offer new insights for colorectal cancer treatment.

## 2. Intestinal flora associated with colorectal cancer development

**Figure 1:**
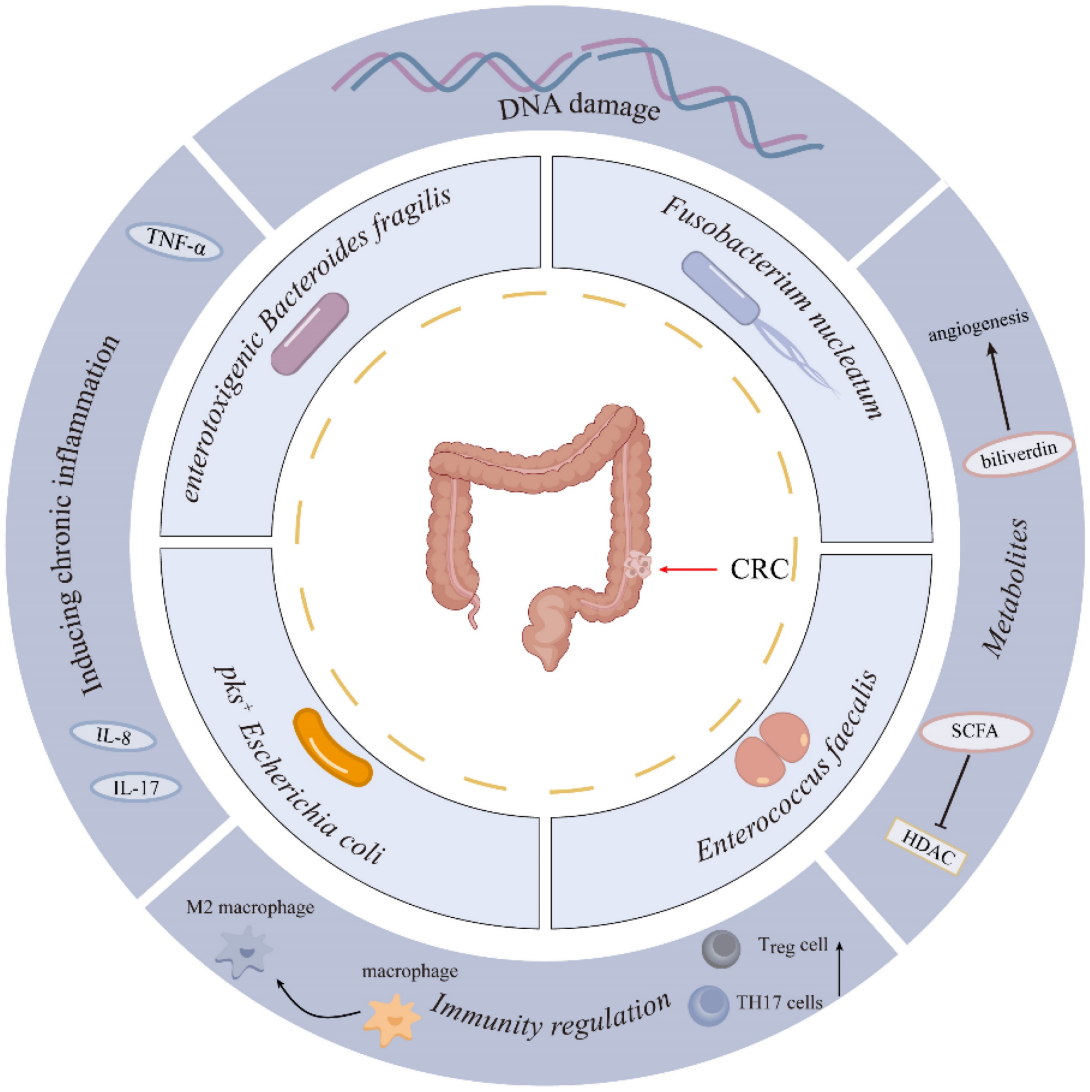
Microbial Species Contributing to Colorectal Carcinogenesis.

Increasing studies have shown a close link between gut microbiota and colorectal carcinogenesis. However, the specific microbial species driving colorectal carcinogenesis, and their causal relationships with CRC initiation/progression, remain to be fully delineated. Advances in genomics and bioinformatics have significantly enhanced the study of bacterial flora. Recent studies highlight the roles of *Fusobacterium nucleatum (F. nucleatum)*, enterotoxigenic *Bacteroides fragilis (B. fragilis)*, *pks^+^ Escherichia coli* (*E. coli*), and *Enterococcus faecalis* (*E. faecalis*) in colorectal cancer development^17^-^19^ (Figure 1). This section summarizes recent studies on the intestinal flora associated with colorectal cancer (Table 1).

### 2.1 Fusobacterium nucleatum

*Fusobacterium nucleatum* is a Gram-negative anaerobic bacterium that primarily colonizes the oral cavity and acts as a conditionally pathogenic organism. Early studies on* F. nucleatum*—a common member of the oral microbiota—focused on its role in oral diseases, particularly its pro-inflammatory effects and impact on immune cell function, which are closely linked to periodontitis and oral tumor progression. However, with the progress of research, the contribution of *F. nucleatum* to colorectal cancer has attracted attention. A 2012 study first observed that *F. nucleatum* signals were enriched in tumor tissues compared to adjacent normal tissues^29^, and its abundance increased as colorectal cancer progressed from early to advanced stages^30^. The abundance of *F. nucleatum* was significantly correlated with colorectal cancer prognosis, with higher levels associated with poorer outcomes^31^, ^32^. Specifically,* F. nucleatum*-high cases showed a 58% increased risk of CRC-specific mortality compared with *F. nucleatum*-negative cases^33^. *F. nucleatum* accumulates in CRC tissues via binding of its Fap2 protein to Gal-GalNAc residues on tumor cell surfaces^34^. *F. nucleatum* proteins FadA promote colorectal cancer by binding to E-cadherin, respectively, activating β-catenin signaling and enhancing tumor proliferation^35^, ^36^. *F. nucleatum* has also been shown to activate the NF-κB pathway in colorectal cancer cells via the ALPK1 receptor, inducing ICAM1 expression and enhancing cancer cell invasiveness and metastasis^37^. *F. nucleatum* can promote colorectal cancer liver metastasis via the miR-5692a/IL-8 axis by inducing epithelial-mesenchymal transition^38^. *F. nucleatum* upregulates integrin α5 (ITGA5) in colorectal cancer by activating E-cadherin/KLF4 signaling in a Ca²⁺-dependent manner. This process enhances tumor growth and metastasis, which can be attenuated by targeting ITGA5 or KLF4^39^.

### 2.2 Enterotoxigenic *Bacteroides fragilis*

*Bacteroides fragilis* is a common Gram-negative bacillus, classified into enterotoxigenic and non-toxin-producing strains. Enterotoxigenic *B. fragilis* secretes a 20-kDa metalloprotease toxin, known as *B. fragilis* toxin. This strain can disrupt the intestinal barrier, promoting inflammation and disease progression^40^, ^41^. A strong association between enterotoxigenic *B. fragilis* and colorectal cancer has also been reported^42^-^44^. When *B. fragilis* colonizes the colon, it produces large amounts of toxins that damage the intestinal mucosa and activate STAT3 in the epithelial cells. STAT3 activation is closely linked to inflammation, cell proliferation, angiogenesis, and cancer development and metastasis. Long-term activation of STAT3 by enterotoxigenic *B. fragilis* maintains a pro-carcinogenic inflammatory environment in colorectal cells, significantly increasing their likelihood of becoming cancerous^45^. Enterotoxigenic *B. fragilis* secretes toxins that cleave E-cadherin on colonic cells, disrupting epithelial cell connections. This damage promotes bacterial translocation and activates the Wnt/β-catenin signaling pathway, which contributes to colorectal carcinogenesis^46^. Furthermore, enterotoxigenic *B. fragilis* activates Toll-like receptor 4 (TLR4) in colorectal cancer cells, upregulating JMJD2B expression through the TLR4-NFAT5-dependent signaling pathway. This results in high NONAG expression and the acquisition of tumor stem cell characteristics^47^.

### 2.3 pks^+^ Escherichia coli

*Escherichia coli*, a common commensal bacterium in the human intestinal tract, includes certain strains capable of causing disease under specific conditions. Analysis of colorectal cancer tissue samples has revealed a significant enrichment of *E. coli* within tumor tissues, with its abundance correlating with cancer stage and prognosis. Notably, some *E. coli* strains harbor polyketide synthase (*pks*) gene islands, which encode colibactin—a genotoxic small molecule that interacts with DNA, inducing damage through its molecular warhead structure^25^, ^48^. The prevalence of *pks*⁺ *E. coli* is higher in CRC patients compared to healthy individuals, suggesting a potential role in tumorigenesis^49^. Emerging evidence indicates that *pks*⁺ *E. coli* promotes CRC development by inducing DNA damage, cell cycle arrest, chromosomal aberrations, and cellular senescence in colorectal cells^50^, ^51^.

### 2.4 Enterococcus faecalis

*Enterococcus faecalis* is the predominant enterococcal species in the human gut, colonizing from the neonatal period and playing a crucial role in intestinal development. In neonates, *E. faecalis* exhibits anti-inflammatory properties and supports colonic maturation by inducing intestinal epithelial cells to secrete IL-10, thereby suppressing inflammation and reducing IL-8 production. Due to its immunomodulatory effects, *E. faecalis* has been utilized in the treatment of chronic sinusitis, bronchitis, and acute diarrhea in children. However, its role in colorectal cancer remains controversial^52^. Some studies suggest that *E. faecalis* may exert protective effects against CRC, as evidenced by the *E. faecalis* EC-12 strain's ability to inhibit β-catenin signaling and suppress tumorigenesis in colorectal cells^53^. Conversely, other studies report a higher abundance of *E. faecalis* in CRC patients compared to healthy individuals, implicating a potential pro-tumorigenic role^54^. *E. faecalis* promotes cell proliferation and angiogenesis in CRC via producing biliverdin. Biliverdin can significantly increase the expression levels of IL-8 and VEGFA by regulating the PI3K/AKT/mTOR signaling pathway^55^. Moreover, *E. faecalis* has been shown to generate reactive oxygen species, leading to colonic DNA damage, genetic instability, and CRC progression^56^. Further research is needed to elucidate the precise role of *E. faecalis* in CRC development.

### 2.5 Other strains of bacteria

In addition to *Fusobacterium nucleatum*, enterotoxigenic *Bacteroides fragilis*, *pks^+^ Escherichia coli*, and *Enterococcus faecalis*, other bacterial strains are also associated with colorectal carcinogenesis, such as *Campylobacter jejuni*. This bacterium produces cytolethal distending toxin (CDT), which are homologous to DNA enzymes and can induce DNA double-strand breaks, leading to gene mutations and chromosomal aberrations that promote colorectal cancer development^57^. Furthermore, the use of rapamycin inhibits the tumor-promoting activity of *Campylobacter jejuni*^58^*. Peptostreptococcus anaerobius* interacts with α2/β1 integrins on colorectal cancer cells via the surface protein PCWBR2. This interaction selectively enriches on the mucosal surface of colorectal cancer, activating the PI3K-Akt signaling pathway and significantly enhancing the proliferative capacity of colorectal cancer cells^26^. The composition of the intestinal flora is diverse, and the interactions between different strains and between strains and the human body are complex. Further studies are needed to elucidate the relationship between common strains and colorectal carcinogenesis.

## 3. Mechanisms of colorectal cancer occurrence and development caused by intestinal flora

**Figure 2:**
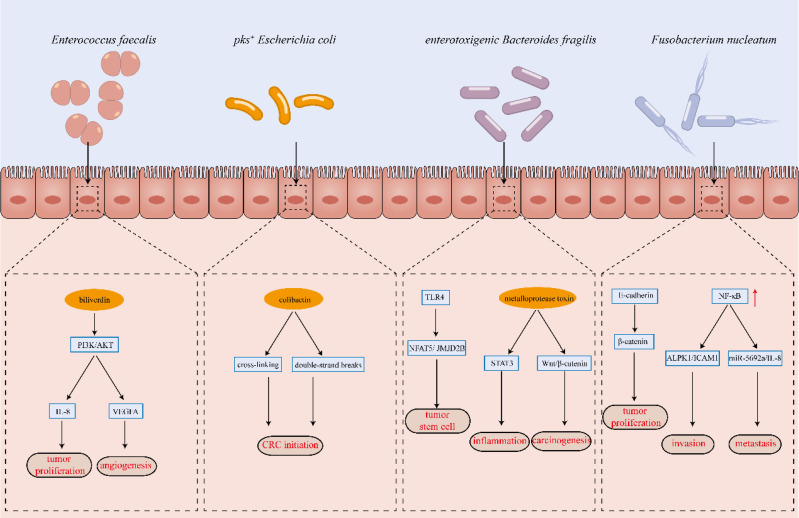
Key Mechanisms of Gut Microbiota Implicated in CRC Development.

The development of colorectal cancer is a multifactorial process influenced by genetic, environmental, dietary, and lifestyle factors. Its initiation and progression result from the complex interplay of these elements. The critical role of the gut microbiota in CRC pathogenesis is well-established; however, the precise mechanisms through which microbial communities contribute to colorectal carcinogenesis remain incompletely understood and are an active area of investigation. This section provides an overview of the key mechanisms implicated in CRC development based on current research findings (Figure 2).

### 3.1 Direct action leading to DNA damage

Colorectal cancer is driven by the accumulation of mutations in proto-oncogenes and oncogenes, with the "adenoma-carcinoma sequence" model describing chromosomal instability as a key feature of disease progression. In this model, genetic mutations lead to hyperplasia and dysplasia of the colonic epithelium, ultimately resulting in malignant transformation. Emerging evidence suggests that specific bacterial strains within the gut microbiota contribute to CRC development by inducing DNA damage in colorectal epithelial cells^59^. For instance, co-culture of *Fusobacterium nucleatum* with CRC cells leads to significant DNA damage and upregulation of the DNA repair factor Chk2^60^. Similarly, *Escherichia coli* harboring *pks*⁺ alkylate adenine residues in DNA, causing double-strand breaks and cross-linking^61^. Whole-genome sequencing of colonic organoids exposed to colibactin has revealed distinct mutational signatures, further supporting the role of *pks*⁺ *E. coli* in colorectal carcinogenesis^62^. Additionally, both *pks*⁺ *E. coli* and enterotoxigenic *Bacteroides fragilis* induce 8-oxoguanine DNA lesions, which are closely linked to CRC initiation^63^. Toxins secreted by enterotoxigenic *B. fragilis* upregulate spermine oxidase in colonic cells, promoting ROS production and subsequent DNA damage^64^. Likewise, *Campylobacter jejuni* produces toxins with DNase activity, leading to DNA double-strand breaks, gene mutations, and chromosomal aberrations, thereby contributing to CRC progression^65^.

### 3.2 Inducing chronic inflammation

Chronic inflammation is a well-established risk factor for tumorigenesis, particularly in colorectal cancer. Persistent intestinal inflammation and poorly controlled inflammatory bowel disease significantly elevate the risk of colorectal carcinogenesis. Pro-inflammatory cytokines such as TNF-α, IL-8, and IL-17 play a crucial role in CRC development and progression^66^. Gut microorganisms contribute to intestinal inflammation by interacting with pattern recognition receptors via surface-associated molecular signatures, triggering the secretion of inflammatory mediators through innate immune signaling pathways. Enterotoxigenic *Bacteroides fragilis* promotes colonic inflammation by activating STAT3 in colonic epithelial cells and inducing IL-17 production. Additionally, its toxin activates the NF-κB pathway via E-cadherin in intestinal epithelial cells, leading to excessive IL-8 secretion and inflammation^67^. Similarly, *Peptostreptococcus anaerobius* has been shown to disrupt the intestinal barrier, and promote macrophage pyroptosis and IL-1β secretion via the TLR2/4-NF-κB-NLRP3 signaling pathway^68^. While intestinal inflammation is a natural component of tissue repair following microbial dysbiosis, chronic and unresolved inflammation can create a pro-tumorigenic environment, thereby increasing the risk of CRC development.

### 3.3 Influence of intestinal metabolites

The gut microbiota colonizes the human intestine and generates a diverse array of metabolites that directly interact with the host, playing a critical role in colorectal cancer initiation and progression^69^. *Enterococcus faecalis* produces biliverdin, which alleviates cell cycle arrest in CRC cells, thereby promoting proliferation and colony formation. Additionally, biliverdin induces angiogenesis and accelerates tumor progression by activating the PI3K/AKT/mTOR signaling pathway, leading to the upregulation of IL-8 and VEGFA in CRC cells^55^. Bile acids, another key class of microbial metabolites, are synthesized as primary bile acids in hepatocytes and subsequently converted into secondary bile acids by the intestinal microbiota, particularly under a high-fat diet^70^. Secondary bile acids, including deoxycholic acid, lithocholic acid, taurolithocholic acid, and their derivatives, exhibit pro-tumorigenic effects by inducing ROS formation, causing DNA damage and gene mutations, disrupting mitosis, and activating the EGFR and NF-κB pathways^71^. Additionally, sulfate-reducing bacteria in the gut metabolize intestinal sulfate into hydrogen sulfide, which induces DNA damage, oxidative stress, inflammation, and colonic mucosal hyperproliferation, thereby promoting CRC development^72^. In contrast, dietary fiber metabolism by gut microbiota produces short-chain fatty acids (SCFAs), such as acetate, propionate, and butyrate, which exert beneficial effects. Among these, butyrate possesses potent anti-inflammatory and antitumor properties by inhibiting histone deacetylase (HDAC), a key regulator of oncogenic gene expression. A decline in butyrate-producing bacteria, such as *Clostridium butyricum* and *Faecalibacterium prausnitzii*, has been associated with an increased risk of CRC^73^.

### 3.4 Regulation of the body's immunity

The immune system plays a crucial role in defending against pathogens and surveilling malignant cells, eliminating cancerous tissues. However, tumor cells can evade immune detection by altering their molecular phenotypes, secreting immunosuppressive cytokines, and recruiting regulatory immune cells. Intestinal microbiota influence cytokine expression and activate specific immune cell populations, thereby modulating both local and systemic immune responses to tumors. The abundance of *F. nucleatum* is inversely correlated with CD3^+^ T cell infiltration in colorectal tumors^74^. Through activation of the NF-κB pathway, *F. nucleatum* upregulates miR-1322 in colorectal cancer cells, leading to increased CCL20 secretion and the induction of M2 macrophage polarization^75^. M2 macrophages, which suppress T cell-mediated antitumor immunity via Arg-1 expression, are closely linked to tumor proliferation, metastasis, and angiogenesis^76^. In colorectal cancer patients with microsatellite instability, *F. nucleatum* abundance is strongly associated with immune responses. Tumors with high *F. nucleatum* levels exhibit increased proliferation, invasiveness, and distinct immune microenvironment alterations, including reduced FoxP3^+^ T-cell infiltration and enhanced M2 macrophage polarization, which collectively impair immunotherapy efficacy^77^. Enterotoxigenic* Bacteroides fragilis* activates STAT3 signaling in colonic mucosal immune cells, upregulating IL-17A and promoting the infiltration of pro-tumorigenic Th17 cells^23^. Moreover, *Streptococcus bovis* stimulates colorectal cancer cells to secrete cytokines such as IL-6, Scyb1, Ptgs2, IL-1β, TNF, and CCL2, thereby recruiting CD11b^+^ TLR-4^+^ immune cells to the tumor site, establishing an immunosuppressive microenvironment that fosters colorectal cancer progression^78^ .* Peptostreptococcus anaerobius* promotes colorectal cancer progression and resistance to anti-PD-1 therapy by activating integrin α2β1-NF-κB signaling to recruit CXCR2^+^ myeloid-derived suppressor cells (MDSCs) and directly enhancing MDSC immunosuppressive activity via lytC_22-Slamf4 interactions^28^.

## 4. Clinical application and treatment strategies

### 4.1 Early screening and diagnosis of colorectal cancer

Colorectal cancer is often asymptomatic in its early stages, leading to late-stage diagnoses and poor prognoses. Early screening is crucial for timely intervention, significantly reducing mortality and improving patient survival rates^79^. Currently, the fecal occult blood test (FOBT) and colonoscopy are the primary screening methods for CRC, and their combined use has been shown to reduce CRC-related mortality by 16%^80^. However, FOBT has limited specificity, necessitating confirmatory colonoscopy for positive cases. Although colonoscopy remains the gold standard due to its high detection accuracy, its invasiveness, high cost, and associated risks, such as perforation and hemorrhage, limit its widespread acceptance and feasibility for large-scale population screening. Therefore, there is an urgent need for a non-invasive, highly sensitive, and specific screening method to enhance early CRC detection and improve clinical outcomes.

The intestinal flora in colorectal cancer patients differs significantly from that in healthy individuals, with a notable increase in strains associated with carcinogenesis and a decrease in protective strains. This suggests that intestinal flora could serve as an early marker for colorectal cancer screening^81^. Recent studies have explored the use of intestinal microorganisms to distinguish colorectal cancer patients from healthy individuals. *F. nucleatum* is significantly enriched in colorectal cancer tissues, detected in 74% of cases, whereas its abundance in peri-tumoral tissues is much lower—about 1/250 of that in cancerous tissues^82^. In colorectal cancer patients, detection rates of pks^+^ *Escherichia coli* and enterotoxigenic *Bacteroides fragilis* are also significantly higher than in healthy individuals, suggesting that *F. nucleatum*, pks^+^ Escherichia coli, and enterotoxigenic *Bacteroides fragilis* could serve as potential biomarkers for colorectal cancer screening^83^. In colorectal cancer screening, testing for *F. nucleatum* abundance combined with fecal immunochemical test (FIT) offers similar specificity but a 26% increase in sensitivity compared to FIT alone. A recent meta-analysis found that *F. nucleatum* had a sensitivity of 71%, specificity of 76%, and an AUC of 0.80 for diagnosing colorectal cancer, indicating its potential as a biomarker for non-invasive screening^84^.

Beyond the microbiome itself, microbial metabolites also hold promise as potential biomarkers for colorectal cancer screening. Notably, short-chain fatty acids, which possess anticancer properties, are significantly reduced in the feces of CRC patients compared to healthy individuals^85^. Moreover, alterations in the levels of amino acids such as proline and cysteine have been observed in CRC samples^86^. A fecal metabolomic analysis using gas chromatography-mass spectrometry further revealed decreased levels of fructose, linoleic acid, and niacin, alongside elevated concentrations of proline and uridine in CRC patients^87^. These findings highlight the potential of fecal metabolites as non-invasive biomarkers for CRC detection, offering new avenues for early diagnosis and screening.

In conclusion, the composition of gut microbiota differs significantly between colorectal cancer patients and healthy individuals, with distinct pathogenic bacterial signatures associated with disease progression. Integrating gut microbial screening—a rapid and non-invasive diagnostic approach—with existing colorectal cancer screening methods can improve both sensitivity and specificity, offering a promising strategy for early detection and intervention.

### 4.2 Influence of intestinal flora on clinical treatment efficacy

Colorectal cancer is currently managed through a multimodal approach that includes surgical intervention, chemotherapy, radiotherapy, immunotherapy, and other treatment modalities^88^-^91^. Given the significant relationship between gut microbiota and colorectal cancer development, as well as their impact on treatment outcomes, there has been growing interest in researching this connection. Numerous studies have demonstrated that gut microbiota can influence the efficacy of chemotherapy, radiotherapy, and immunotherapy in the treatment of colorectal cancer^92^, ^93^.

#### 4.2.1 Chemotherapy

Intestinal flora can influence the efficacy of chemotherapeutic drugs by regulating their metabolism in colorectal cancer cells. In colorectal cancer patients treated with 5-fluorouracil after radical surgery, the abundance of *Fusobacterium nucleatum* is correlated with chemoresistance. Further studies demonstrated that *Fusobacterium nucleatum* upregulated the expression of BIRC3 via the TLR4/NF-κB signaling pathway, which directly inhibited apoptosis by suppressing the cysteine asparaginase cascade, thereby contributing to drug resistance in colorectal cancer cells^94^. *Fusobacterium nucleatum* can also inhibit the expression of miR-18a and miR-4802 via the TLR4/MYD88 innate immune signaling pathway, leading to increased expression of ULK1 and ATG7, which activate autophagy and contribute to drug resistance to oxaliplatin and 5-fluorouracil in colorectal cancer cells^95^. In addition to influencing drug resistance by affecting drug metabolism and response, some bacterial strains can directly alter chemotherapeutic drugs, rendering them inactive and reducing their antitumor efficacy. Literature reports indicate that in the presence of specific *γ-Proteobacteria* or *Escherichia coli* in tumors, gemcitabine is converted into its inactive form by cytidine deaminase, reducing its anticancer efficacy^96^.

Intestinal flora not only influences chemotherapy efficacy but also plays a significant role in chemotherapy-related adverse effects. Approximately 30% of chemotherapy patients experience chemotherapy-related pain. Chemotherapy-induced peripheral neuropathy causes neuropathic pain that can persist for months or even years, limiting chemotherapy dosages and hindering optimal therapeutic outcomes. Intestinal flora plays a crucial role in chemotherapy-induced mechanical pain hypersensitivity; oxaliplatin-induced hypersensitivity was reduced in germ-free mice and in mice pretreated with antibiotics. This is mainly due to the interaction between bacterial LPS and TLR4 on macrophages, which stimulates the secretion of inflammatory factors in response to oxaliplatin, leading to mechanical pain hypersensitivity^97^, ^98^. Irinotecan, a DNA topoisomerase I inhibitor, blocks DNA replication and RNA synthesis. It is a first-line treatment for advanced colorectal cancer but causes serious gastrointestinal side effects, including mucositis and delayed diarrhea. *In vivo*, irinotecan is converted to SN38, which inhibits DNA topoisomerase I and tumor proliferation. SN38 is then cleared via the gastrointestinal tract by binding to glucuronic acid, forming the inactive SN38-G. However, β-glucuronidase produced by intestinal commensal bacteria removes glucuronic acid from SN38-G, reactivating SN38 and causing intestinal epithelial damage and hemorrhagic diarrhea. In the human gut, β-glucuronidase is primarily expressed by *Enterococcus faecalis*. Diarrhea caused by irinotecan can be prevented by selectively inhibiting this enzyme, allowing for dose intensification and improving irinotecan effectiveness^99^.

#### 4.2.2 Radiotherapy

Radiotherapy can rapidly and persistently alter the composition of the intestinal flora, increasing the abundance of *Bacteroides* and decreasing *Clostridium* in the intestines of mice treated with systemic radiotherapy compared to controls^100^. A study found that vancomycin treatment, which alters the Gram-positive flora in the intestinal microbiota, significantly enhanced both the direct and distant antitumor effects of radiotherapy by remodeling the tumor microenvironment and promoting antigen presentation in the draining lymph nodes^101^. Beyond influencing tumor sensitivity to radiotherapy, intestinal flora also impacts radiotherapy toxicity. Germ-free mice receiving lethal whole-body irradiation showed reduced endothelial and lymphocyte apoptosis in the small intestinal villi compared to conventionally reared mice, and were significantly more resistant to radiation enteritis. This resistance was linked to the Fiaf factor, a fibrinogen/angiopoietin-like protein typically secreted by small intestinal villous epithelial cells, but inhibited by intestinal flora. Numerous studies have shown that modulating the specific microbial composition of the gut through flora transplantation can either exacerbate or alleviate intestinal radiation damage^102^, ^103^.

#### 4.2.3 Immunotherapy

The advent of immunotherapy has revolutionized cancer treatment, yielding remarkable therapeutic success across multiple malignancies. Immunotherapy is primarily indicated for patients with microsatellite instability-high (MSI-H) and mismatch repair-deficient (dMMR) CRC. With its growing application, the impact of gut microbiota on immunotherapy efficacy has garnered increasing attention. Clinical studies have demonstrated a strong correlation between gut microbiome composition and the response to immune checkpoint inhibitors (ICIs)^104^, ^105^. Preclinical models further support these findings, showing that increasing the abundance of *Lactobacillus rhamnosus* GG in the gut enhances dendritic cell and CD8^+^ T-cell infiltration into colorectal tumors. This bacterium activates the cGAS/STING signaling pathway in dendritic cells, induces IFN-γ secretion, and potentiates the efficacy of PD-1 blockade therapy^106^. Additionally, *Bifidobacterium pseudolongum, Lactobacillus johnsonii*, and *Olsenella* have been shown to improve ICI response in various murine cancer models, with the gut microbiota-derived metabolite inosine playing a pivotal role in immune activation^107^. *Fusobacterium nucleatum* promotes colorectal cancer by inducing an ALPK1-dependent pro-inflammatory response and upregulating PD-L1 expression^108^. Moreover, a clinical study investigating regorafenib combined with toripalimab in metastatic colorectal cancer revealed that patients with high *F. nucleatum* abundance had a lower immunotherapy response rate and shorter median progression-free survival compared to those with lower *F. nucleatum* levels^109^.

### 4.3 Interventions for intestinal flora

Intestinal flora significantly impacts the efficacy of chemotherapy, radiotherapy, and immunotherapy in colorectal cancer. Modulating the composition of the gut flora can influence the effectiveness of CRC therapies, making this approach a potential therapeutic strategy^110^.

Fecal microbiota transplantation (FMT) involves transferring the gut microbial community from a donor to a patient^111^. This approach reduces competitive inhibition in the recipient's microbiome while enhancing overall diversity and stability, offering advantages over targeting the abundance of a single microbial species. FMT is widely used to treat *Clostridioides difficile (C. difficile)* infections and inflammatory bowel disease^112^, ^113^. While FMT has shown promise in enhancing the efficacy of ICI in melanoma patients^114^, conclusive data on FMT's efficacy in colorectal cancer clinical trials is lacking. Some preclinical studies demonstrate that transplanting feces from colorectal cancer patients into mice elevated intestinal inflammatory factors and increased the occurrence of high-grade dysplasia and polyps, indicating that the microbiota of colorectal cancer patients promotes carcinogenesis in animal models^115^. Conversely, transplanting gut microbiota from healthy mice increased resistance to carcinogen-induced colorectal cancer in recipient mice^116^. Although FMT's safety and efficacy in treating *C. difficile* infections are well-documented, potential risks such as pathogen transmission remain, and its use in immunocompromised colorectal cancer patients is still controversial.

Diet is one of the most significant factors affecting gut microbial composition, with a direct correlation between gut flora and dietary substrates. The gut microbiota rapidly alters in response to dietary modifications^117^. Dietary fiber modifies gut microbiota composition by enhancing the abundance of probiotics like *Bifidobacteria* and *Lactobacillus*, while also increasing butyrate levels, which possess anticancer properties, via microbial fermentation^118^. In contrast, red meat and processed meat consumption is linked to a higher risk of colorectal cancer, classified as carcinogenic, and reducing red meat and processed meat intake can significantly lower colorectal cancer incidence^119^, ^120^. Modulating patients' intestinal flora through dietary interventions seems to be a safe, feasible, and cost-effective approach^121^. However, if a healthy diet cannot be sustained following dietary changes, gut microbiota composition may revert to its original state. Changing long-term dietary habits is challenging and difficult to monitor, highlighting the need for scientifically sound and easily implementable dietary regimens.

Probiotics are microorganisms that confer health benefits, and when administered in adequate amounts, they can restore the balance of intestinal flora and enhance overall health. Preclinical studies indicate that genera such as *Bifidobacterium* and *Lactobacillus* spp. can exert tumor-suppressive effects by inhibiting cell proliferation, inducing apoptosis in tumor cells, enhancing antitumor immunity, and producing anticancer compounds^122^. However, questions regarding which probiotic strains to use for treating colorectal cancer, the optimal ratios of each strain, the appropriate dosages, and potential pitfalls remain unresolved.

Prebiotics (e.g., inulin, fructo-oligosaccharides) represent another critical intervention, as they selectively stimulate the growth of beneficial taxa (e.g., Bifidobacterium spp.) to restore gut microbial homeostasis. Recent trials have shown prebiotic supplementation reduces CRC-associated inflammation markers^123^, supporting their potential as adjuvant therapies.

## 5. Summary and Outlook

The critical role of intestinal flora in colorectal carcinogenesis, progression, and treatment has been recognized and validated by previous studies. The identification of colorectal cancer-associated pathogenic bacteria and metabolic markers, along with the elucidation of flora-host interaction mechanisms, contributes to early screening, diagnosis, and treatment of colorectal cancer, offering novel insights into innovative diagnostic and therapeutic approaches.

Differences in gut flora between colorectal cancer patients and healthy individuals highlight their potential as screening markers, and advances in microbiological testing technology have facilitated their clinical application. Although some studies have shown promising results by combining gut flora testing with existing colorectal cancer screening techniques, leading to significant improvements in sensitivity and specificity, challenges related to reproducibility and standardization persist. These challenges arise from the complexity of the intestinal microbiota and the influence of environmental and genetic factors. Designing an optimal combination of biomarkers and validating them across diverse populations to develop a safe and cost-effective clinical screening technique remains a significant research challenge.

Numerous methods can influence the composition of gut flora, many of which have been explored in clinical trials, including probiotics, prebiotics, antibiotics, FMT, dietary modifications, and physical activity. However, the optimal approach to manipulate the microbiota remains to be determined. It remains unclear which method—fecal microbiota transplantation, dietary modification, or probiotic intervention—is superior. The criteria for utilizing FMT and probiotic supplementation in colorectal cancer patients, along with potential contraindications, are still under investigation. Further research is necessary to identify which strains are effective for clinical use, the optimal administration rates, and appropriate dosages. Individual differences in probiotic colonization within the intestinal mucosa exist, highlighting the need for comprehensive analysis of a patient's microbiome, metabolome, and dietary factors. This approach can facilitate the design of individualized treatments through microbial modification.

Although numerous studies have demonstrated an association between intestinal flora and colorectal cancer, research on fungi, viruses, and protozoa—non-bacterial components of the intestinal microbiota—remains limited. This gap is primarily due to the low abundance of these non-bacterial components in the intestinal tract and the challenges associated with their detection and investigation. The gut microecology represents a complex and balanced system in which each component interacts with one another. Focusing exclusively on the bacterial components may lead to a one-sided understanding, leaving many questions regarding the roles of non-bacterial components unresolved.

Given the vast number and complex, variable composition of intestinal flora, elucidating the relationship between gut microbiota and the occurrence and progression of colorectal cancer remains a challenging task. Advances in sequencing genomics, bioinformatics analysis technologies, and cultivation techniques may lead to breakthroughs in future research. Integrative multi-omics approaches (e.g., metagenomics + metabolomics + transcriptomics) can be used to construct robust gut microbial diagnostic models for early CRC, while machine learning algorithms can optimize personalized FMT regimens by predicting patient response based on baseline microbial composition^124^, ^125^.

## Figures and Tables

**Table 1:** Pathogenic Gut Bacteria in Colorectal Cancer Development

Classification	Bacteria	Influence	Mechanisms
*Fusobacteriota*	*Fusobacterium nucleatum*	Adheres to the epithelium; modulates immune response; promotes inflammation	Activates β-catenin signaling via FadA binding to E-cadherin, leading to upregulation of Cyclin D1^20^; Suppresses immune cytotoxicity via Fap2-TIGIT interaction; Induces a stem-like phenotype and chemoresistance^21^
*Bacteroidota*	*Enterotoxigenic Bacteroides fragilis*	Produces *B. fragilis* toxin (BFT); Disrupts epithelial barrier; Triggers Th17 inflammation	BFT cleaves E-cadherin and activates β-catenin signaling^22^; Induces IL-17-mediated inflammation^23^; Activates STAT3 in epithelial cells, leading to upregulation of ZEB2^24^
*Proteobacteria*	*pks^+^ Escherichia coli*	Produces colibactin; Induces DNA double-strand breaks	Colibactin alkylates host DNA^25^, causes genomic instability and specific mutation signatures; Promotes carcinogenesis
*Firmicutes*	*Enterococcus faecalis*	Produces reactive oxygen species (ROS); Induces DNA damage and macrophage activation	Secretes superoxide that causes DNA strand breaks; Promotes tumor-associated inflammation via COX-2/PGE2
*Campylobacterota*	*Campylobacter jejuni*	Adheres to mucosa; Secretes cytolethal distending toxin	CDT induces DNA damage and cell cycle arrest; Disrupts epithelial integrity; Promotes IL-8-driven inflammatory response
*Firmicutes*	*Peptostreptococcus anaerobius*	Alters lipid metabolism; Activates TLR2/4 signaling	Activates PI3K-Akt pathway via α2/β1 integrin, leading to increased cell proliferation^26^; Enhances ROS production and cholesterol biosynthesis^27^; Facilitates tumor-promoting microenvironment^28^

## Abbreviations

*B. fragilis*: *Bacteroides fragilis*
*C. difficile*: *Clostridioides difficile*
CRC: colorectal cancer
CDT: cytolethal distending toxin
*E. faecalis*: *Enterococcus faecalis*
*E. coli*: *Escherichia coli*
FIT: fecal immunochemical test
FMT: fecal microbiota transplantation
FOBT: fecal occult blood test
*F. nucleatum*: *Fusobacterium nucleatum*
HDAC: histone deacetylase
ICIs: immune checkpoint inhibitors
ITGA5: integrin α5
MyD88: myeloid differentiation primary response 88
MDSCs: myeloid-derived suppressor cells
MSI-H: microsatellite instability-high
dMMR: mismatch repair-deficient
pks: polyketide synthase
ROS: reactive oxygen species
SCFAs: short-chain fatty acids
TLR4: Toll-like receptor 4
